# Fibroblastic niches in action: *CCL19*^+^ reticular cells drive anti-tumor immunity in lung cancer

**DOI:** 10.1038/s41392-025-02185-z

**Published:** 2025-03-24

**Authors:** Alessandro Ianni, Alejandro Vaquero, Thomas Braun

**Affiliations:** 1 Department of Cardiac Development and Remodeling, Max-Planck-Institute for Heart and Lung Research, 61231 Bad Nauheim, Germany; 2 Chromatin Biology Laboratory, Josep Carreras Leukaemia Research Institute (IJC), 08916 Badalona, Spain

**Keywords:** Tumour immunology, Lung cancer

In a groundbreaking study published in *Cell*, Onder and colleagues revealed the pivotal role of fibroblastic reticular cells (FRCs) in creating specialized niches, providing robust immune activation against non-small cell lung cancer (NSCLC), which paves the way for innovative therapeutic strategies.^1^

Cancers are complex ecosystems comprising tumor cells intricately interwoven with a diverse array of non-cancerous cells—including immune cells, cancer-associated fibroblasts (CAFs), endothelial cells, and various tissue-specific resident cells—embedded within a remodeled extracellular matrix (ECM), collectively shaping the tumor microenvironment (TME).^2^ The TME differs markedly among tumors, influenced by factors such as tumor location, stage, the inherent properties of the cancer cells, and individual patient conditions. The TME plays a crucial role in cancer progression by regulating processes such as cancer cell migration, proliferation, tumor vascularization, and the modulation of anti-cancer immune responses.^2^ Effects of the TME are partially mediated by secreted factors, including exosomes, metabolites, cytokines, chemokines, and a remodeled extracellular matrix produced by CAFs, which profoundly influence cancer progression.^2^ Numerous studies revealed that the TME plays a pivotal role in modulating anti-cancer immune responses, either promoting or suppressing these processes in a manner that appears highly context-dependent.^3^

Effective immune responses against cancer cells basically involve three different key mechanisms. (i) tumor-antigen-specific CD8^+^ T cells are activated in draining lymph nodes and then (ii) recruited into the tumor. (iii) Recruited CD8^+^ T cells are maintained in an activated state within specialized niches in the TME.^1^ The TME controls these mechanisms through multiple pathways. First, its cellular components secrete immune modulators that regulate CD8^+^ T cell activity and migration. Second, the TME’s physical and metabolic landscape may either facilitate or impede infiltration and survival of immune cells. A notable example of these interactions is the role of CAFs in shaping immune responses.^3^

**Fig. 1:**
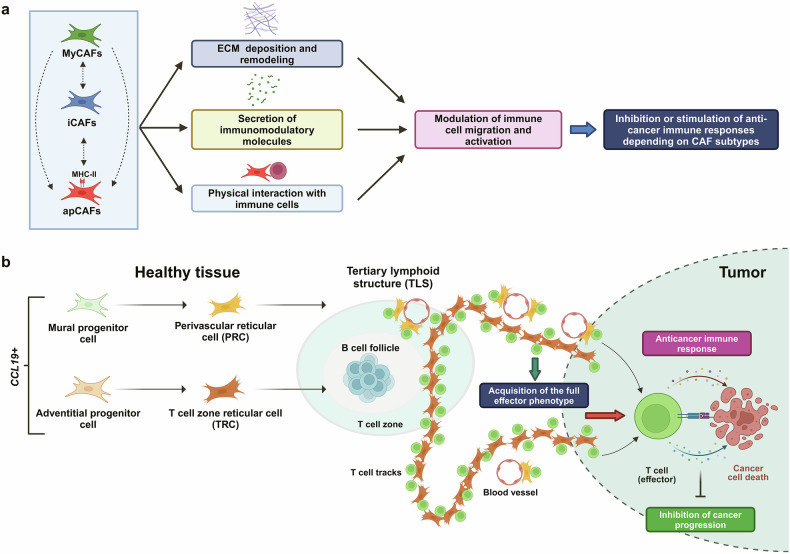
The complex role of cancer-associated fibroblasts (CAFs) and fibroblastic reticular cells (FRCs) in regulating anti-cancer immune responses**. a** CAFs represent a highly heterogeneous population broadly categorized into three main subsets: myofibroblastic CAFs (myCAFs), inflammatory CAFs (iCAFs), and antigen-presenting CAFs (apCAFs). These cells originate from diverse sources and exhibit significant plasticity, allowing interconversion between subtypes in response to different stimuli (dashed arrows). CAFs modulate anti-cancer immune responses through various mechanisms: (i) remodeling the extracellular matrix (ECM) to influence immune cell migration within the tumor, (ii) secreting immunomodulatory molecules, and (iii) in the case of apCAFs, by physically interacting with immune cells *via* antigen presentation through the major histocompatibility complex class II (MHC-II). Notably, CAFs can exert both immunosuppressive and immunostimulatory effects, indicating the presence of distinct subpopulations that drive these opposing functions.^3^ **b** In lung cancer, two primary subpopulations of FRCs, perivascular reticular cells (PRCs) and T-zone reticular cells (TRCs), arise from mural and adventitial *CCL19*-expressing progenitor cells in healthy lung tissue, following distinct differentiation pathways. FRCs play a pivotal role in the formation of specialized lymphoid structures, such as tertiary lymphoid structures (TLSs), located at the tumor periphery, as well as T-cell racks that connect TLSs and extend into the tumor parenchyma. By physically interacting with CD8^+^ T cells, FRCs promote the acquisition of a fully activated effector phenotype, enhancing anti-cancer responses and thereby restraining lung cancer progression.^1^ This figure was created using BioRender (www.biorender.com)

CAFs have different origins, including tissue-resident fibroblasts and mesenchymal stem cells. Their heterogeneity and dynamic interconversion of different subtypes allow for both stimulatory and inhibitory effects on cancer immunity.^3^ Based on transcriptional profiles and functions, CAFs have been broadly categorized into three main types: myofibroblastic CAFs (myCAFs), inflammatory CAFs (iCAFs), and antigen-presenting CAFs (apCAFs). Some CAF subtypes, such as myCAFs, form dense stroma, blocking T cell migration and suppressing their activity, whereas others secrete immunomodulators, influencing cancer immunity positively or negatively as extensively reviewed elsewhere.^3^ Identification of subpopulations of CAFs with specific roles in cancer immunity is crucial for reprogramming them into subtypes that promote anti-cancer immune responses (Fig. 1).^3^

Onder and collaborators made substantial progress in this direction by identifying a specific CAF subset that is indispensable for shaping specialized tumor niches, including tertiary lymphoid structures (TLSs) and T cell tracks within the TME. TLSs, located at the tumor margin, are complex immune aggregates primarily composed of B cells and other immune populations, playing a critical role in regulating immune responses. TLSs are connected with the tumor bed through T cell tracks that build specialized physical conduits.^1^ The composition and frequency of TLSs have emerged as key predictors for the success of immunotherapies, highlighting their role in enhancing anti-tumor T cell responses. Prior to this study, cells responsible for establishing such niches in lung cancer remained largely enigmatic.^1^

Using single-cell transcriptomic analysis, Onder et al. identified two main clusters of *CCL19*-expressing CAFs in human non-small cell lung cancer (NSCLC), resembling the transcriptional profiles of perivascular reticular cells (PRCs) and T-cell zone reticular cells (TRCs), previously characterized in lymphoid organs.^1^ These fibroblastic reticular cells (FRCs) are a specialized subset of stromal cells within immune-regulating structures. They play a critical role in immunoregulation by facilitating immune cell migration and activation in response to specific biological needs. For instance, *CCL19*-expressing TRCs guide T cells into the parenchyma of lymph nodes and the splenic white pulp in collaboration with dendritic cells, sustaining T-cell immune reactivity. Additionally, TRCs and B-cell zone reticular cells (BRCs) are interconnected with the vasculature through perivascular reticular cells (PRCs), which facilitate immune cell trafficking and contribute to the overall regulation of immune responses.^4^ Onder and colleagues revealed that *CCL19*-expressing FRCs are essential for establishing specialized TLSs and T-cell tracks in NSCLC. Their findings demonstrated that these FRCs predominantly interact with CD8^+^ T cells within the TME, orchestrating the formation of fibroblastic niches that sustain T-cell activity (Fig. 1).^1^

Consistent with this finding, in vivo ablation of *Ccl19*^+^ cells in the lung using the diphtheria toxin receptor system disrupted the interconnections of FRCs and CD8^+^ T cells in the TME of mice immunized with a recombinant coronavirus expressing a tumor-specific antigen. Single-cell transcriptomic analysis revealed that in vivo depletion of *Ccl19*^+^ cells diminished expression of genes associated with CD8^+^ T-cell differentiation, activation, and proliferation, while increasing the expression of exhaustion markers, which contrasts sharply with what was observed in FRCs-competent mice. The study underscores the pivotal role of *Ccl19*^+^ FRCs in enhancing T-cell effector functions within the TME in NSCLC and suggests that CD8^+^ T cells achieve full effector potential through their interactions with *CCL19*-expressing FRCs in the TME after initial activation and proliferation in draining lymph nodes.^1^

Remarkably, ablation of *Ccl19*^+^ FRCs in the lung substantially increases the tumor burden in xenograft models of lung cancer in immunized mice, underscoring the critical role of these cells for sustaining robust anti-cancer immune responses and slowing lung cancer progression (Fig. 1).^1^

In this work, the authors made substantial progress in uncovering the cellular origin of FRCs. Single-cell transcriptomics and lineage tracing in mice conclusively demonstrated that FRCs in the TME arise from *Ccl19*-expressing mural and adventitial progenitors in healthy lung tissue, which give rise to PRCs and TRCs, respectively (Fig. 1).^1^ Remarkably, rather than aligning strictly with any of the established CAF categories, FRCs exhibit unique transcriptomic signatures. They express genes associated with all three main CAF subsets,^1^ suggesting that they represent a distinct subpopulation within the broader CAF landscape.

As our understanding of TRC and PRC subsets in regulating CD8^+^ T cells deepens, including identification of chemokines driving their differentiation from precursor cells, reprogramming these cells into immune-supportive populations holds transformative potential for lung cancer treatment. This strategy may redefine cancer immunomodulation, moving beyond the current reliance on immune checkpoint blockade (ICB) therapies, which are limited by variable patient responsiveness and significant side effects linked to the immunological traits of specific cancers. Targeting the TME’s immunological landscape offers an exciting path for pioneering next-generation cancer immunotherapies.^5^
